# Vasopressin Contributes to Respiratory and Cardiovascular Regulation in Spontaneously Hypertensive and Normotensive Rats

**DOI:** 10.3390/jcm14228019

**Published:** 2025-11-12

**Authors:** Michał Proczka, Adam Trzciński, Agnieszka Cudnoch-Jędrzejewska, Jacek Przybylski, Tymoteusz Żera

**Affiliations:** 1 Department of Experimental and Clinical Physiology, Laboratory of Centre for Preclinical Research, Medical University of Warsaw, 02-091 Warsaw, Poland; michal.proczka@wum.edu.pl (M.P.); lek.adam.trzcinski@gmail.com (A.T.); agnieszka.cudnoch-jedrzejewska@wum.edu.pl (A.C.-J.); 2 Department of General, Vascular, Endocrine and Transplant Surgery, Medical University of Warsaw, 02-091 Warsaw, Poland; 3 Department of Biophysics, Physiology, and Pathophysiology, Medical University of Warsaw, 02-091 Warsaw, Poland; jacek.przybylski@emeritus.wum.edu.pl

**Keywords:** antidiuretic hormone, blood pressure, chemoreflex, hypertension, vasopressin, vasopressin 1a receptor (V1aR), ventilation

## Abstract

**Background**: Vasopressin (AVP) and its V1a receptor (V1aR) are involved in the regulation of the cardiovascular system. Limited evidence suggests that AVP may also contribute to respiratory regulation. Arterial chemoreflex is the main reflex involved in cardiorespiratory regulation and is triggered from the carotid bodies (CBs), specialized organs that detect changes in arterial blood content. Both increased activity of the vasopressinergic system and enhanced arterial chemoreflex have been found in hypertension. Here, we aimed at determining cardiorespiratory responses to AVP in normo- and hypertensive rats and the involvement of CBs and V1aRs. **Methods**: Experiments were performed in urethane-anesthetized adult male spontaneously hypertensive (SHR) and normotensive Wistar Kyoto (WKY) rats. Arterial blood pressure (MABP), heart rate (HR), femoral artery blood flow (FABF), minute ventilation (MV), respiratory rate (RR), and end-tidal carbon dioxide (ETCO2) were recorded. We evaluated cardiorespiratory responses to arterial chemoreflex activation with potassium cyanide, intravenous AVP, V1aR antagonist, and CB denervation. **Results**: In comparison to normotensive animals, SHR rats had significantly greater resting MABP, HR, MV, and enhanced pressor and ventilatory components of arterial chemoreflex. CB denervation caused insignificant changes in cardiorespiratory parameters. Intravenous administration of AVP resulted in a significant increase in MABP in both groups, which was greater in SHR rats, and in ventilatory inhibition, which was present only in SHR rats. CB denervation reduced the pressor response to AVP in normotensive rats and abolished the inhibitory effect of AVP on ventilation in SHR rats. Intravenous administration of the V1aR antagonist caused a significantly greater decrease in MABP in the hypertensive group. Only SHR rats responded with an increase in ventilation after the V1aR antagonist. Effects of AVP were abolished after blockade of V1aRs in both groups. **Conclusions**: Our study indicates that (i) SHR rats show augmented cardiorespiratory response to AVP, (ii) cardiorespiratory effects of AVP depend on V1aRs; and (iii) respiratory effects of AVP in the hypertensive rats appear to be primarily mediated by CBs.

## 1. Introduction

Vasopressin (AVP) is a key neurohormone involved in adapting body functions to homeostatic disturbances. In addition to the renal antidiuretic effects, AVP regulates arterial blood pressure, sympathetic activity, baroreflex sensitivity, glucose metabolism, release of glucocorticoids and catecholamines, stress response, and thermoregulation [1,2,3]. Vasopressin is synthesized in paraventricular and supraoptic nuclei of the hypothalamus and is then transported to the posterior lobe of the pituitary gland, where it is released into the circulation in response to increased plasma osmolality [1,4,5,6]. Vasopressin is also released by non-osmotic stimuli, including hypovolemia, hypotension, hypoglycemia, strenuous exercise, angiotensin II, and hypoxia [1]. Vasopressin influences the cardiovascular system mainly by acting through vasopressin type 1a receptors (V1aRs), which cause vasoconstriction and an increase in vascular resistance in most of the vascular beds, as well as affect coronary circulation and cardiac hemodynamics [3,7]. The cardiovascular system efficiently supplies tissues with oxygen only if working in concert with the respiratory system, which depends on reciprocal interactions between the two [8]. Thus, AVP has also been suggested to be engaged in the regulation of the respiratory system both as a blood-borne neurohormone and as a neurotransmitter within the central nervous system [9]. There are conflicting results regarding the role of V1aR in the respiratory centers of the medulla, with both excitatory and inhibitory effects reported by different investigators [10,11,12,13]. Increased plasma concentration of copeptin, which is a biomarker of AVP release, has been found in circulatory and respiratory disturbances, including hypertension, myocardial infarction, heart failure, valvular heart disease, hypovolemia, hypotonia, hypoxemia, severe pneumonia, SARS-CoV-2 infection, pulmonary embolism, and brain trauma [1,14,15,16,17,18].

Carotid bodies (CBs) contain chemosensitive cells that are mainly stimulated by low partial pressure of oxygen but are also responsive to high carbon dioxide partial pressure, increased blood acidity, hypoperfusion, and hypoglycemia [19]. Stimulation of the chemoreceptors within the CBs evokes the peripheral chemoreflex that leads to cardiovascular and respiratory responses, including sympathoexcitation, hyperventilation, changes in the heart rate, and arousal [19,20,21,22]. Several neuropeptides, hormones, and vasoactive mediators, such as endothelin 1 (ET-1) or glucagonlike peptide 1 (GLP-1), have been found to modulate CB function [23,24,25,26]. Increased sensitivity and tonicity of the peripheral chemoreflex drives sympathoexcitation with resultant high blood pressure [27], a phenomenon that has been found in hypertensive animals [28,29,30,31,32] and humans [33,34,35]. Modulation of CB activity has also been postulated as a means of alleviating hyperactivity of the sympathetic nervous system in hypertension and heart failure [27,36]. Upregulation of the vasopressinergic system has also been suggested to play an important role in primary hypertension [1]. It was found that SHR rats manifest stronger pressor responses to intravenous [37] and intracerebroventricular [38] administrations of AVP. Higher plasma AVP concentrations are associated with an increase in arterial blood pressure [37]. SHR rats were also shown to have an enhanced AVP release from the pituitary gland in comparison to WKY rats [39]. In addition, we found that V1aRs are expressed in the CBs [40] and CB chemosensitive cells express G protein q/11 and phosphokinase C, which constitute key intracellular components of V1aR signaling [41]. Thus, CBs are a plausible target for AVP present in the bloodstream both under physiological conditions and in disease states associated with elevated plasma AVP concentration. In the current study, we evaluated cardiorespiratory responses to intravenously administered AVP and compared whether the responses differ in normo- and hypertensive animals. We also assessed the role of CBs and V1aRs in mediating AVP-induced cardiorespiratory effects under physiological and hypertensive conditions.

## 2. Materials and Methods

### 2.1. Animals

The study was performed on male normotensive Wistar Kyoto (WKY) (*n* = 12) and spontaneously hypertensive (SHR) rats (*n* = 12), weighing between 310 and 360 g. The rats were obtained from the breeding facility at the Medical University of Warsaw, Poland. Animals were housed 3 per cage with a 12:12 h light/dark cycle and with access to a standard rat pellet diet and tap water ad libitum. Prior to the surgical procedures, animals were anesthetized with intraperitoneal urethane injection (1.5 g/kg, Sigma-Aldrich, (Merck KGaA), Darmstadt, Germany). Urethane anesthesia was shown to have minimal effect on cardio-respiratory parameters as well as cardiovascular reflexes and was used previously in similar studies [42,43,44]. The study was approved by the II Local Ethics Committee for Animal Experimentation at the Warsaw University of Life Sciences. The experiments were carried out in accordance with domestic regulations and Directive 2010/63/EU of the Council of 22 September 2010 on the protection of animals used for scientific purposes.

### 2.2. Surgical Procedures

*Intravascular catheters*. Vascular catheters were implanted as described previously [45]. In brief, the femoral neurovascular bundle was exposed on the left side. Vascular polyurethane catheters (intravascular part—Cat # BB520-25; extravascular part—BB520-40; Scientific Commodities, Inc., Lake Havasu City, AZ, USA) were filled with heparinized saline (unfractionated heparin 500 IU/mL; Polfa Warszawa SA, Warsaw, Poland) and implanted into the femoral artery and the femoral vein. Then, the catheters were secured with a suture. The tip of the arterial catheter was located in the descending aorta below the branching of the renal arteries, and the tip of the venous catheter was placed in the inferior vena cava. The arterial and venous catheters served to record pulsatile blood pressure and for intravenous administration of the examined substances, respectively.

*Doppler probe.* Recording of the arterial blood flow was performed as described previously [46]. The incision was made in the right groin, and the femoral neurovascular bundle was exposed. The right femoral artery was carefully separated from surrounding tissue and the femoral vein; a Doppler probe was placed on the artery. The measurements were performed with the use of a transit time ultrasound set-up, which consists of a non-cannulating acoustic (20 kHz) flow probe connected to a dedicated flowmeter (type T106, Transonic System Inc., Ithaca, NY, USA).

*ECG electrodes.* Two stainless steel needle electrodes were placed subcutaneously in the left ventral area of the thorax and in the right lower quadrant of the abdomen for bipolar recording of electrocardiogram (ECG) and heart rate (HR).

*Carotid body denervation.* In a subset of animals, both carotid bifurcations were carefully dissected. In some of the animals, both sinus nerves were carefully exposed from the surrounding tissue and selectively cut under a surgical microscope to permanently inhibit chemoreflex as previously described [28,47,48]. Successful CB denervation was confirmed by the absence of ventilatory response and attenuation or loss of the pressor response to i.v. administration of potassium cyanide (KCN) (30 μg/100 μL; Sigma-Aldrich, (Merck KGaA), Darmstadt, Germany) [28].

*Tracheal tube.* Finally, the trachea was exposed rostrally from the sternum. An approximately 3 mm horizontal incision of the annular ligament at mid-level between the sternum and larynx was performed. A polyethylene tubing (internal diameter—2 mm; external diameter—2.5 mm; length—15 mm) was implanted into the trachea and secured with a suture placed around the trachea. Next, the flow head dedicated to small laboratory animals was tightly connected to the tracheal tube (RX237B, Biopac Systems, Goleta, CA, USA) for recording of the airflow and respiratory rate (RR) [40]. A rodent capnograph (CapnoScan, Kent Scientific, Torrington, CT, USA) was also connected to the air outflow for recording of the end-tidal carbon dioxide (ETCO2), as described previously [49].

### 2.3. Hemodynamic and Ventilatory Measurements

**Figure 1:**
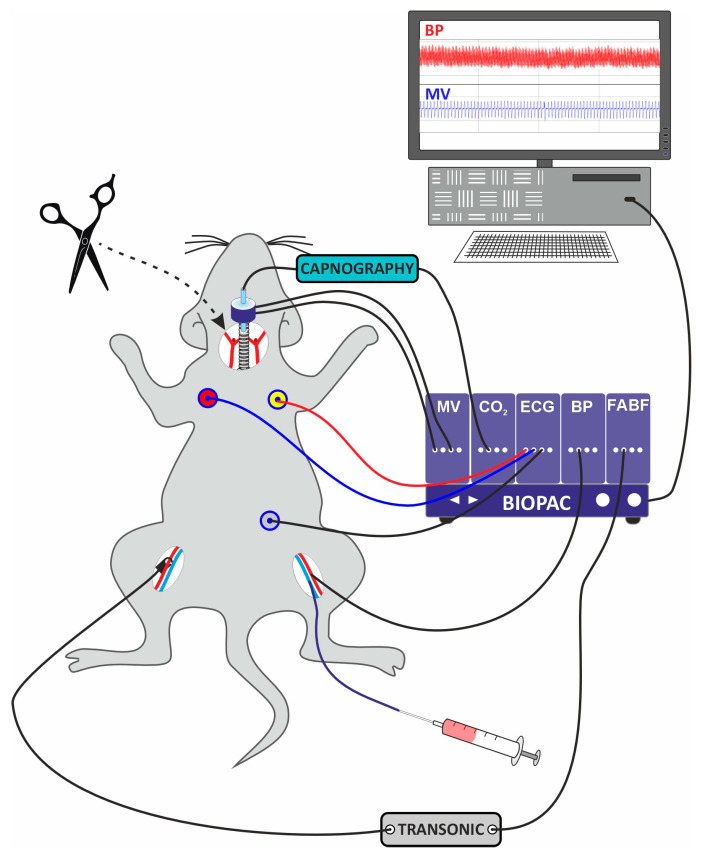
Schematic representation of a rat prepared for recording of circulatory and respiratory parameters. Connected: tracheal tube to the airflow head and differential pressure transducer for minute ventilation (MV); capnography for end-tidal CO_2_ (ETCO2); arterial catheter to the pressure transducer for blood pressure (BP); venous catheter to a micro syringe for intravenous administrations; Doppler probe to flowmeter (Transonic) for femoral artery blood flow (FABF); electrodes for electrocardiography (ECG). The site (scissors) of bilateral carotid body denervation/sham surgery is marked.

After calibration of the recording apparatus, the blood pressure transducer was connected to the arterial catheter, and the differential pressure transducer to the airflow head. Blood pressure, airflow, and ECG signals were amplified and fed into an analog-digital converter, digitized, and recorded on the PC station (MP100 system, Biopac Systems, Goleta, CA, USA). Signals for femoral artery blood flow (FABF) and capnography were fed into the MP100 rig and recorded simultaneously with blood pressure and airflow. All signals were sampled at 1 kHz. Using the AcqKnowledge 3.7 software (Biopac Systems, Goleta, CA, USA), the following parameters were derived: mean arterial blood pressure (MABP) from the pulsatile blood pressure, heart rate from the ECG, mean FABF from the arterial flow, minute ventilation (MV) and respiratory rate (RR) from the airflow, and ETCO2 from the capnography. The scheme showing the rat prepared for the cardio-respiratory measurements is shown in Figure 1.

### 2.4. Experimental Protocols of the Hemodynamic and Ventilatory Parameters

*Protocol 1.* Ten minutes were allowed for stabilization of cardio-respiratory parameters. Baseline hemodynamic and respiratory parameters were recorded in all studied animals (SHR: *n* = 12, WKY: *n* = 12). Then, after 10 min, the chemoreflex was triggered with i.v. administration of potassium cyanide (KCN) (30 μg/100 μL). After stabilization of hemodynamic and respiratory parameters (10 min), AVP was administered (10 ng/100 μL i.v.; Sigma-Aldrich, (Merck KGaA), Darmstadt, Germany)). The dose of AVP was chosen based on previously published data that showed a biological effect [40,50,51].

*Protocol 2.* After completing measurements from Protocol 1, half of the animals (SHR: *n* = 6, WKY: *n* = 6) underwent measurements from Protocol 2. Ten minutes were allowed for stabilization of cardio-respiratory parameters. Baseline hemodynamic and respiratory parameters were measured. Carotid bodies were bilaterally surgically denervated; 10 min after CB denervation, a second dose of AVP was i.v. administered.

*Protocol 3.* After completing measurements from Protocol 1, the remaining half of the rats (SHR: *n* = 6, WKY: *n* = 6) underwent measurements from Protocol 3. Ten minutes were allowed for stabilization of cardio-respiratory parameters. Selective V1aR antagonist (d(CH2)51,Tyr(Me)2,Arg8) Vasopressin (Tocris, Bristol, UK) was administered i.v. (5 μg/100 μL). The dose of V1aR antagonist was also based on previously described studies and was effective in completely blocking hemodynamic responses to peripheral AVP administration. After stabilization of hemodynamic and respiratory parameters (10 min), the second i.v. dose of AVP was repeated.

**Figure 2:**
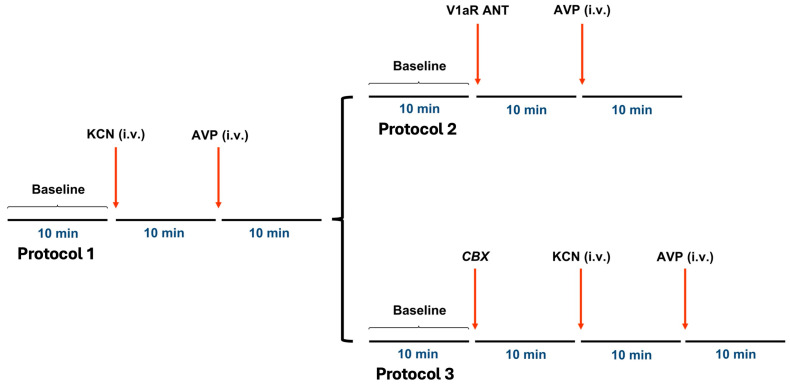
Initially, all rats received intravenous (i.v.) infusions of KCN (30 μg/100 μL) followed by AVP (10 ng/100 μL) (Protocol 1). Subsequently, animals received either i.v. infusion of V1aR ANT (5 μg/100 μL) followed by AVP (10 ng/100 μL) (Protocol 2) or after carotid body denervation received i.v. infusions of KCN (30 μg/100 μL) and AVP (10 ng/100 μL) (Protocol 3).

All substances were given as a bolus. The protocol of the study is shown in Figure 2.

### 2.5. Statistical Analysis

Statistical analysis was carried out in Statistica 13.3 (StatSoft Inc., Tulsa, OK, USA). MABP, HR, RR, and MV were averaged over 30 s intervals for baseline values. After KCN administration, a 3 s period of maximal hemodynamic and respiratory responses was used for analysis; 30 s recordings of parameters were analyzed 1 min after KCN, after AVP, after V1aR antagonist administration, as well as after carotid body denervation. FABF and MV were compared and analyzed as a percentage change from the preceding recording, accounting for potential drift in the recording parameters. Significant changes in cardiorespiratory parameters after all previously described administrations were detected with the use of Student’s *t*-tests for dependent samples. Changes in MABP, HR, % MV, RR, % FABF, and ETCO2 were compared with a Student’s *t*-test for independent samples. If the Shapiro–Wilk test was significant, the Wilcoxon test for dependent samples and the nonparametric Mann–Whitney U test for comparison between groups were used. A value of *p* < 0.05 was considered significant.

All data are presented as mean values with standard deviation. If the data did not fulfil a normal distribution, the median value with interquartile range (IQR) is provided. Box plots were generated with a web tool, BoxPlotR [52].

## 3. Results

### 3.1. Resting Cardio-Respiratory Parameters Are Higher in Spontaneously Hypertensive than Normotensive Rats

The resting MABP and HR were significantly higher in hypertensive SHR than in normotensive WKY rats. At rest, there was no significant difference in FABF. The resting MV and RR were respectively higher and lower in hypertensive SHR rats in comparison to normotensive WKY controls. However, there were no significant differences in the ETCO2. All parameters are summarized in Table 1.

### 3.2. Spontaneously Hypertensive Rats Show Greater Sensitivity of Arterial Chemoreflex

**Figure 3:**
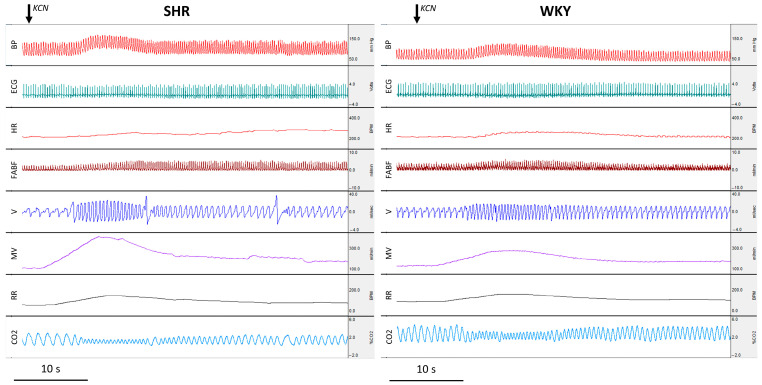
Arterial chemoreflex—hemodynamic and respiratory responses to KCN in spontaneously hypertensive (SHR) rats and normotensive Wistar Kyoto control rats. Cardiorespiratory response is greater in SHR than in WKY rats. Recordings of pulsatile arterial blood pressure (BP), electrocardiogram (ECG), heart rate (HR) derived from ECG, femoral artery blood flow (FABF), airflow (V), minute ventilation (MV), respiratory rate (RR), and capnography (CO_2_).

**Figure 4:**
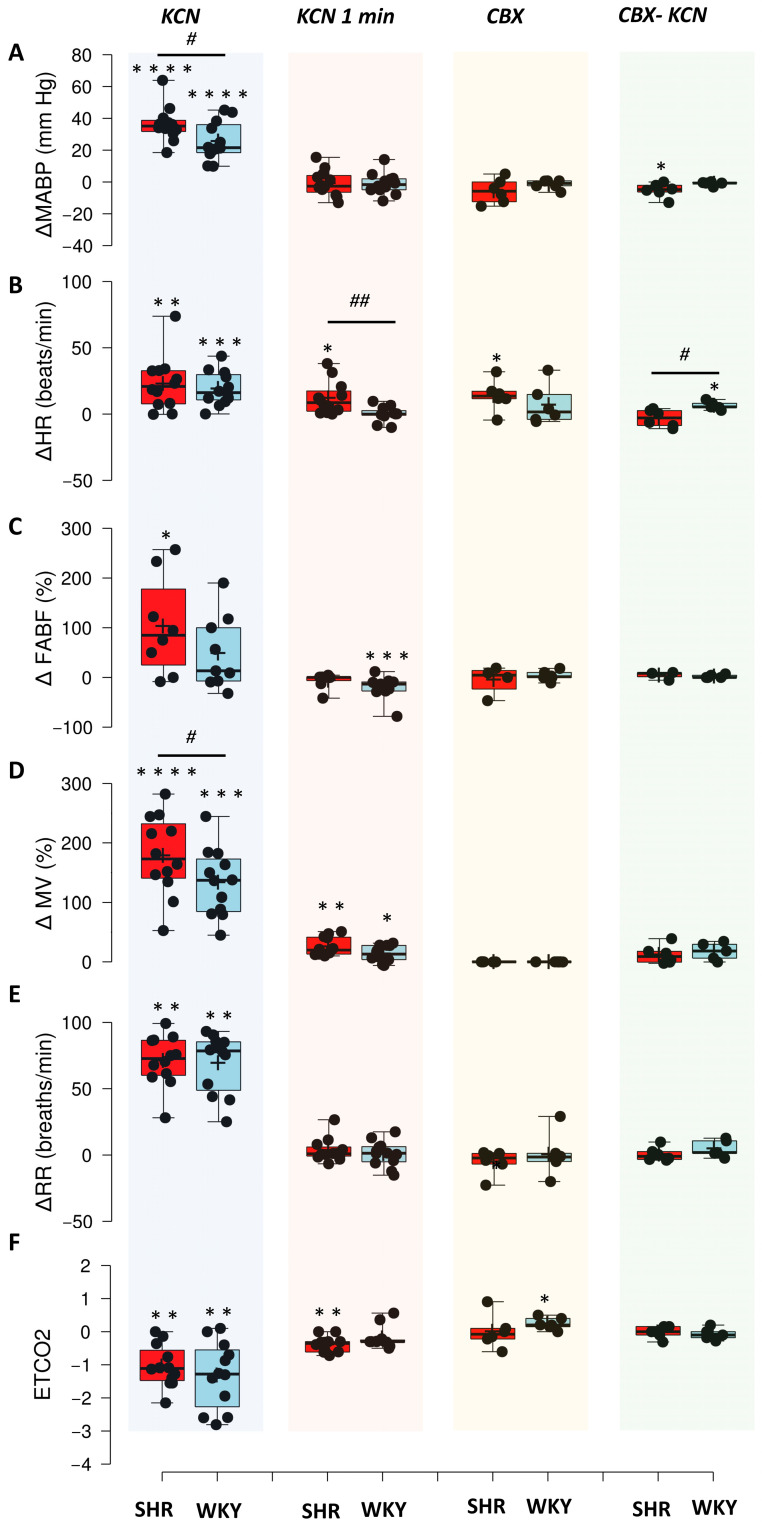
Cardiorespiratory responses to KCN-evoked arterial chemoreflex, 1 min after KCN, carotid body denervation (CBX), and KCN after the CBX. (**A**) change in mean arterial blood pressure (ΔMABP; mm Hg); (**B**) heart rate (ΔHR); (**C**) percentage change in femoral artery blood flow (ΔFABF); (**D**) change in minute ventilation (ΔMV); (**E**) change in respiratory rate (ΔRR); (**F**) change in the end-tidal CO_2_ (ΔETCO2). Boxplots: box—IQR; whiskers—max-min; plus—mean; dash—median. Red squares—SHR rats; blue squares—WKY rats. Dots represent individual data points. * *p* < 0.05, ** *p* < 0.01, *** *p* < 0.001, **** *p* < 0.0001—after vs. before intervention; # *p* < 0.05, ## *p* < 0.01,—SHR vs. WKY.

The raw recordings of hemodynamic and respiratory responses to arterial chemoreflex activation by intravenous KCN in SHR and WKY rats are presented in Figure 3. The increase in MABP in response to pharmacologically triggered arterial chemoreflex with KCN was significant for both SHR and WKY rats (*p* < 0.0001, paired Student’s *t*-test for SHR; *p* < 0.001, paired Student’s *t*-test for WKY rats). However, the change in MABP was significantly greater in the SHR rats in comparison to WKY controls (*p* < 0.05, Student’s *t*-test) (Figure 4A). One minute after initiation of the reflex, MABP returned to the baseline values in both groups. The tachycardic response to KCN, marked by an increase in HR, was significant in both groups (*p* < 0.01, paired Mann–Whitney test for SHR; *p* < 0.001, paired Student’s *t*-test for WKY rats). There was no significant difference in the changes in HR between both groups (Figure 4B). One minute after the reflex, the HR remained significantly increased in the SHR rats in comparison to the resting value (*p* < 0.05, paired Student’s *t*-test for SHR), and it returned to the baseline values in the normotensive WKY rats. There was a significantly greater difference in the HR from the baseline between the SHR and WKY rats (*p* < 0.01, Student’s *t*-test) (Figure 4B). Pharmacologically triggered arterial chemoreflex also resulted in a significant increase in the FABF only in the SHR rats (*p* < 0.05, paired Student’s *t*-test for SHR; *p* < NS, paired Student’s *t*-test for WKY rats); however, there were no significant differences in the changes in FABF between hypertensive SHR and normotensive WKY rats (Figure 4C). Activation of the arterial chemoreflex resulted in a significant increase in MV (*p* < 0.0001, paired Student’s *t*-test for SHR; *p* < 0.001, paired Student’s *t*-test for WKY rats) and RR (*p* < 0.01, paired Mann–Whitney test for SHR; *p* < 0.01, paired Mann–Whitney test for WKY rats), with the change in MV significantly greater in the SHR rats than in WKY control (*p* < 0.05, Student’s *t*-test). The increased MV was also present 1 min after KCN-triggered arterial chemoreflex in both groups (*p* < 0.01, paired Mann–Whitney test for SHR; *p* < 0.05, paired Student’s *t*-test for WKY rats); however, the changes in MV 1 min after the activation of arterial chemoreflex were similar between SHR and WKY rats (Figure 4D,E). In addition, 1 min after the KCN-triggered arterial chemoreflex, changes in RR were insignificant. Increased MV during the reflex was accompanied by a significant decrease in ETCO2 in both groups (*p* < 0.01, paired Mann–Whitney test for SHR; *p* < 0.01, paired Mann–Whitney test for WKY rats), and significantly lower ETCO2 was also present 1 min after the KCN-triggered arterial reflex in the SHR rats (*p* < 0.01, Mann–Whitney test); however, there were no significant differences of ETCO2 change from baseline at 1 min after the reflex between SHR and WKY rats (Figure 4F). Collectively, these results indicate a greater cardio-respiratory response to the arterial chemoreflex activation in SHR rats than in normotensive WKY controls.

The bilateral carotid body denervation (CBX) decreased MABP in SHR rats; however, it did not reach significance (Figure 4A). Furthermore, CBX resulted in a modest increase in HR, which was significant only in the SHR rats (Figure 4B), and it had no effect on FABF (Figure 4C). The effect of CBX on ventilatory parameters was insignificant (Figure 4D,E), except for a slight increase in ETCO2 in WKY rats (Figure 4F). Administration of KCN after the CBX decreased MABP only in SHR rats (*p* < 0.05, paired Student’s *t*-test) (Figure 4A), and increased HR only in WKY rats (*p* < 0.05, paired Student’s *t*-test). The change in HR in response to KCN after CBX was significantly higher in WKY rats than in SHR ones (*p* < 0.05, Student’s *t*-test) (Figure 4B). In addition, KCN administration after CBX had an insignificant effect on other cardio-respiratory parameters (Figure 4C–F). Together, these results are indicative of effective denervation of the carotid bodies.

### 3.3. Spontaneously Hypertensive Rats Show Greater Hemodynamic and Respiratory Response to Vasopressin

**Figure 5:**
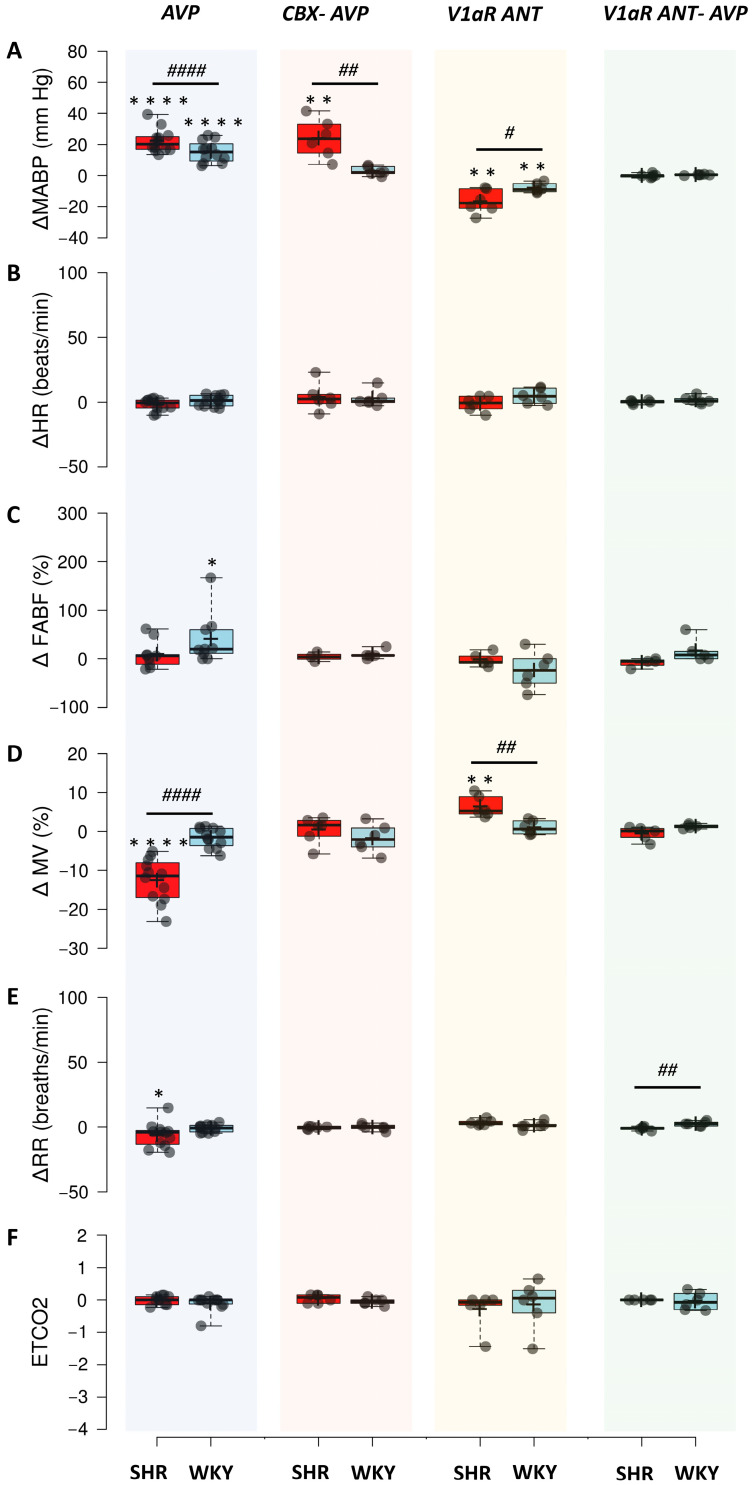
Cardiorespiratory responses to AVP, AVP after carotid body denervation (CBX), V1aR ANT, and AVP after the V1aR ANT. (**A**) change in mean arterial blood pressure (ΔMABP; mm Hg); (**B**) heart rate (ΔHR); (**C**) percentage change in femoral artery blood flow (ΔFABF); (**D**) change in minute ventilation (ΔMV); (**E**) change in respiratory rate (ΔRR); (**F**) change in the end-tidal CO_2_ (ΔETCO2). Boxplots: box—IQR; whiskers—max-min; plus—mean; dash—median. Red squares—SHR rats; blue squares—WKY rats. Dots represent individual data points. * *p* < 0.05, ** *p* < 0.01, **** *p* < 0.0001—after vs. before intervention; # *p* < 0.05, ## *p* < 0.01, #### *p* < 0.0001—SHR vs. WKY.

Intravenous administration of AVP triggered an increase in MABP in both SHR and WKY rats (*p* < 0.0001, paired Student’s *t*-test for SHR rats; *p* < 0.0001, paired Student’s *t*-test for WKY rats). The change in MABP was significantly greater in hypertensive SHR rats than in normotensive controls (*p* < 0.0001, Student’s *t*-test) (Figure 5A). Administration of AVP had an insignificant effect on the HR in both strains of rats (Figure 5B). Furthermore, AVP caused a modest but significant increase in FABP only in the normotensive WKY rats (*p* < 0.05, paired Mann–Whitney test); however, changes in FABP were not significantly different between SHR and WKY rats (Figure 5C). Intravenous administration of AVP caused a significant decrease in MV in SHR rats (*p* < 0.0001, paired Student’s *t*-test for SHR rats) but had an insignificant effect on MV in WKY rats. Furthermore, changes in MV induced by AVP were significantly different between SHR and WKY rats (*p* < 0.0001, Student’s *t*-test) (Figure 5D). Decrease in MV in SHR rats was accompanied by a significant decrease in RR (*p* < 0.05, paired Student’s *t*-test for SHR rats); however, there were no significant differences in changes in HR between SHR and WKY rats (Figure 5E). In addition, changes in ETCO2 were also insignificant (Figure 5F).

Administration of AVP after CBX resulted in a significant increase in MABP in SHR rats (*p* < 0.01, paired Student’s *t*-test), which was comparable to that of the intact SHR rats. However, administration of AVP after CBX resulted in insignificant changes in MABP in normotensive WKY rats (Figure 5A). Furthermore, the change in MABP was significantly smaller after CBX than before CBX in WKY rats (*p* < 0.001, paired Student’s *t*-test). Administration of AVP after CBX had an insignificant effect on HR, FABF, and all respiratory parameters in both SHR and WKY rats (Figure 5B–F). Moreover, there was a significant difference in MV change induced by AVP before and after CBX in SHR rats, with a reduction of MV with intact CBs and a lack of MV changes in CBX rats (*p* < 0.01, paired Student’s *t*-test).

Blockade of V1aRs resulted in a significant decrease in MABP in both hypertensive and normotensive rats (*p* < 0.01, paired Student’s *t*-test for SHR rats; *p* < 0.01, paired Student’s *t*-test for WKY rats). The changes in MABP were significantly different between SHR and WKY rats, with SHR rats showing a greater decrease in MABP (*p* < 0.05, Student’s *t*-test) (Figure 5A). However, administration of V1aR antagonist had an insignificant effect on HR and FABF in both SHR and WKY rats (Figure 5B,C). Blockade of V1aRs resulted in a significant increase in MV only in SHR rats (*p* < 0.01, paired Student’s *t*-test), and changes in MV were significantly greater in SHR rats than in WKY controls (*p* < 0.01, Student’s *t*-test) (Figure 5D). There were no significant changes in RR and ETCO2 in both strains of rats (Figure 5E,F).

Infusion of AVP after administration of V1aR antagonist had an insignificant effect on hemodynamic parameters in both strains of rats. Furthermore, the changes in RR were significantly greater in WKY rats than in SHR (*p* < 0.01, Student’s *t*-test) (Figure 5E). There were no significant changes in ETCO2 after administration of AVP preceded by V1aR antagonist, as well as between strains of rats.

## 4. Discussion

Our study provides evidence for significant respiratory responses to exogenous vasopressin and a distinct pattern of responses in spontaneously hypertensive (SHR) rats from normotensive WKY controls. Specifically, the main results of our study include (1) SHR rats show greater pressor response to intravenous AVP than normotensive WKY rats, (2) this pressor response is accompanied by decrease in pulmonary ventilation only in hypertensive animals, (3) carotid body denervation has insignificant effect on vasopressin pressor effect in SHR; however, it diminishes blood pressure increase in normotensive rats, (4) despite lack of effect on blood pressure increase, CB denervation prevents AVP-induced decrease in pulmonary ventilation in SHR rats, and 5) the cardio-respiratory effects of vasopressin appear to be mediated by V1aRs. In addition, we confirm previously reported increased sensitivity of the arterial chemoreflex in spontaneously hypertensive rats, manifested by greater cardio-respiratory response to the arterial chemoreflex activation.

Spontaneously hypertensive rats are characterized by genetically determined high arterial blood pressure that closely resembles essential hypertension in humans [53]. In our study, the SHR rats manifest a hypertensive phenotype with high blood pressure, which is in line with previous findings both in awake and urethane anesthetized SHR rats [28,30,54]. In addition, we found increased minute ventilation at rest in SHR rats, which is consistent with earlier findings in this strain [55]. In fact, the metabolic rate of SHR rats is higher than in normotensive animals [56,57], and they show a tendency for metabolic acidosis from the early state of pre-hypertension [58]. These results, together with end-tidal carbon dioxide (ETCO2), which was similar in both hypertensive SHR and normotensive WKY rats, indicate that higher pulmonary ventilation in SHR rats should not be interpreted as hyperventilation, as would be indicated by lower ETCO2, but rather is secondary to increased metabolism and production of CO_2_.

The cardiovascular and ventilatory responses to arterial chemoreflex activation are well characterized and reviewed [19,21]. As expected, in our study, hypertensive SHR rats show greater cardiorespiratory response to arterial chemoreflex activation than normotensive WKY. The carotid sinus nerve that innervates the carotid body shows enhanced firing in response to hypoxia or cyanide in SHR rats [29,59], indicative of increased afferent signaling from the carotid body to the cardiovascular centers of the brainstem in this rat strain. Furthermore, increased sensitivity and tonicity of the arterial chemoreflex, characterized by enhanced sympathetic activity and pressor response, have been causally associated with hypertension and have been found across numerous studies in SHR rats [28,29,30,31,32] and hypertensive humans [33,34,35]. In our study, both respiratory and hemodynamic components of the arterial chemoreflex evoked with KCN showed greater magnitude in SHR rats than in WKY controls, suggestive of increased sensitivity of the reflex in hypertensive animals. Denervation of the carotid bodies in our study did not have a significant effect on cardiorespiratory parameters at rest, indicating that in our experimental paradigm with urethane anesthesia, the arterial chemoreflex was not tonically active in hypertensive rats. This finding contrasts with other reports that showed a decrease in sympathetic activity and a fall in arterial blood pressure after denervation or lesioning of the carotid bodies in conscious SHR rats [28,29,60] or deactivation of carotid bodies with 100% oxygen breathing in humans [34]. We also found tachycardic response in both the hypertensive SHR and normotensive WKY rats. Numerous studies, especially in the in situ preparations, report bradycardia during the arterial chemoreflex [61,62,63]. It should be noted that in most of these studies, afferent input from pulmonary stretch receptors is absent. Therefore, the primary arterial chemoreflex response is typically investigated. In experiments involving an intact pulmonary system, such as in our study, afferents from lung mechanoreceptors are activated by breathing. This activation leads to tachycardia [30,64], and the reflex is referred to as the secondary arterial chemoreflex response [19,21]. Furthermore, we found that arterial chemoreflex leads to an increase in femoral artery blood flow, nearly doubling its resting value in hypertensive rats. However, studies investigating blood flow to peripheral organs in response to arterial chemoreflex activation are scarce. Activation of the arterial chemoreflex leads to profound sympathoexcitation with resultant high vascular resistance, enhanced venous return to the heart, and increased cardiac output [19,21]. Since blood flow to an organ depends directly on pressure gradient and inversely on vascular resistance [65,66], we interpret the transiently increased femoral artery blood flow in SHR rats as a result of greater increase in blood pressure than increase in vascular resistance, whereas in normotensive WKY rats lack of changes in femoral artery blood flow as a result of comparable changes in the rise in blood pressure and the increase in vascular resistance.

Activation of the arterial chemoreflex also triggered a respiratory response. In contrast to the decrease in minute ventilation in urethane-anesthetized hypertensive rats with deactivated carotid bodies by breathing 100% oxygen [55], denervation of the carotid bodies in our study did not have a significant effect on cardiorespiratory parameters, indicating that in our experimental paradigm with urethane anesthesia, the arterial chemoreflex was not tonically active in hypertensive rats.

The respiratory component is often evaluated in the in situ preparations by recording the phrenic nerve activity or respiratory rate [67]. However, a limited number of studies reported ventilatory responses in intact rats [40,55,68]. Here, we evaluated the respiratory component in intact animals by recording minute ventilation, respiratory rate, and end-tidal carbon dioxide (ETCO2). Both hypertensive SHR and normotensive WKY rats showed increased pulmonary ventilation and respiratory rate with accompanying significant decrease in ETCO2, indicative of hyperventilation during the reflex. Furthermore, hypertensive SHR rats showed significantly greater increase in ventilation than normotensive controls, suggestive of enhanced ventilatory response to arterial chemoreflex activation in hypertension. This aligns with human studies that showed increased ventilatory response to arterial chemoreflex activation in young hypertensive patients [35] and in hypertensive patients who experienced blood pressure following unilateral carotid body removal [33].

It should be noted that some parameters were still altered at 1 min after the reflex activation. Specifically, SHR rats still showed increased minute ventilation, decreased ETCO2, and elevated heart rate, whereas normotensive WKY rats showed some modestly higher minute ventilation and slightly decreased femoral artery blood flow. These changes maintained at 1 min after KCN administration further point to increased sensitivity of the arterial chemoreflex in the hypertensive SHR rats. Jointly, our findings confirm increased sensitivity of the arterial chemoreflex in spontaneously hypertensive rats.

We found that hypertensive SHR rats show greater cardiorespiratory response to vasopressin. Specifically, the pressor response to intravenous AVP was significantly greater in hypertensive than in normotensive rats, a phenomenon earlier reported [37]. In this line, enhanced sympathoexcitatory and pressor activity of the brain vasopressinergic system was also found in SHR rats [38]. Furthermore, inhibition of V1aRs with a selective blocker lowered arterial blood pressure only in SHR rats, which is in line with previous findings [69], suggesting the tonic activity of the vasopressinergic system is involved in maintaining resting blood pressure in hypertensive SHR rats. We did not find significant changes in the heart rate in response to AVP infusion despite a robust rise in arterial blood pressure and expected slowing of the heart rate due to activation of the arterial baroreflex. We attribute this weak chronotropic response to the fact that urethane anesthesia is known to selectively decrease the gain of the cardiac (chronotropic) component of the baroreflex, thus limiting the chronotropic response to changes in arterial blood pressure [70]. Collectively, our results and previously published findings indicate that the vasopressinergic system is enhanced in spontaneously hypertensive rats.

Besides its renal effects, vasopressin’s pressor effect in the regulation of the cardiovascular system is well recognized [3,7]. Recently, we have postulated that vasopressin may act as a neurohormone that contributes to adjusting the respiratory system under disturbances of homeostasis or stress conditions [9]. One of the likely targets for AVP in the bloodstream is the carotid body. We have found that chemosensitive glomus cells in carotid bodies obtained from the normotensive Sprague-Dawley rats show the presence of immunostaining for both N-terminal and C-terminal fragments of the V1aR [40]. Moreover, intracellular components of V1aR signaling, such as G protein q/11, have been found in the chemosensitive glomus cells in the mouse [41]. Therefore, circulating AVP may affect the activity of the carotid body by binding to V1aRs in the glomus cells. In addition to direct effects on the chemosensitive glomus cell, it is possible that AVP may decrease blood flow to the carotid body via its vasoconstrictor action. Reduced carotid body blood flow sensitizes the arterial chemoreceptors [71,72]. However, the direct effects of AVP on the carotid body artery and carotid body blood flow are not known. In this light, AVP may cause vasodilation of cerebral arteries [73] and distension of the carotid artery in a V1R-dependent manner [74]. Thus, further experiments with selective down-regulation of V1aRs in glomus cells or the carotid body vasculature should address the potential mechanisms by which AVP interacts with carotid bodies. Another plausible site for detecting circulating AVP is the circumventricular organs (CVOs) of the brain that lack the blood–brain barrier and are readily accessed by substances present in the blood [9,50]. Available evidence indicates that CVOs, including the organum vasculosum laminae terminalis (OVLT), the subfornical organ (SFO), and the area postrema (AP), predominantly express V1aRs [11,75,76,77,78,79]. Finally, the AVP-induced increase in arterial blood pressure activates the arterial baroreflex [80].

Inhibitory effects on pulmonary ventilation of intravenously infused AVP were found in awake and anesthetized rats [40,81,82,83], conscious dogs [84], and fetal lambs [85]. At least in part, this can be interpreted as the arterial baroreflex-mediated inhibition of ventilation [86,87,88,89]. In our previous study, administration of AVP in normotensive Sprague-Dawley rats resulted in a decrease in minute ventilation; however, the rise of blood pressure in those rats was significantly higher than in the current report (~40 mm Hg vs. ~20 mm Hg) [40]. It should be noted that despite the rise in arterial blood pressure in both the SHR and WKY rats, AVP administration was associated with a significant decrease in minute ventilation and respiratory rate only in the hypertensive animals. It was shown that stimulation of baroreceptors, on the contrary to chemoreceptors, inhibits respiratory drive [87,88]. Therefore, increased activity of baroreceptors caused by blood pressure rise is the most plausible explanation for the respiratory effects of vasopressin administration in SHR. However, the baroreflex is blunted in SHR rats in comparison to normotensive animals [29,30,60]; thus, it is also possible that vasopressin exerts its respiratory effects beyond the interactions of baroreflex with the respiratory system and carotid bodies or that the sensitivity of the respiratory regulation to AVP is dependent on specific rat strains. This notion is further corroborated by an altered pattern of cardiorespiratory response to AVP after denervation of the carotid bodies. Specifically, carotid body denervation had an insignificant effect on the pressor action of AVP in hypertensive SHR rats, but the changes in blood pressure were significantly lower in normotensive WKY rats. In addition, despite a similar increase in arterial blood pressure to the conditions with intact carotid bodies, AVP had an insignificant effect on minute ventilation in SHR rats with denervated carotid bodies. These observations suggest that AVP interacting with carotid bodies may contribute to the pressor response to AVP in normotensive animals, but its CB-mediated respiratory effects are mainly present in hypertensive SHR rats. The reason for the qualitative difference in respiratory effects of vasopressin between SHR and WKY clearly calls for further research.

SHR rats showed a greater cardiorespiratory response to administration of V1aR antagonist than normotensive WKY controls, with significantly increased pulmonary ventilation in hypertensive SHR rats. Similar transient effects of V1aR blockade on pulmonary ventilation were found in awake dogs [90]. The inhibitory effects of V1aRs have also been revealed by studies, in which V1aR antagonists unmask stimulatory effects on the respiratory activity of oxytocin in rats [83] and Ang II in dogs [91].

In contrast to SHR rats, the effect of V1aR antagonist administration on respiratory parameters was insignificant in normotensive WKY rats, which is in line with findings previously reported by us in urethane-anaesthetized normotensive Sprague-Dawley rats [40] and by Walker and Jennings, who found an insignificant effect of V1aR blockade on pulmonary ventilation in conscious normotensive rats [92].

In our study, both respiratory and hemodynamic effects of AVP were found to be mediated by V1aRs, as a selective antagonist of the receptor effectively abolished all responses to intravenously administered AVP. These findings are in line with previously published studies that show that systemic blockade of V1aRs with selective antagonists prevents changes in pulmonary ventilation and arterial blood pressure induced by intravenous administration of AVP in anesthetized rats [40,81,83]. Our results further corroborate available evidence indicating that effects of AVP on regulation of respiratory and cardiovascular systems are predominantly dependent on V1aRs [3,9,93]. It should be noted that in our study, both AVP and V1aR antagonist were administered intravenously. Therefore, V1aRs in the circulatory and respiratory systems were activated by AVP and blocked by the antagonist, respectively. In addition, carotid bodies express V1aRs [9,40], so peripherally administered AVP or the antagonist should also target vasopressin receptors located in the carotid body. Thus, carotid body denervation allowed for the separation of systemic effects of AVP from local carotid body-dependent responses triggered by AVP in our study. Furthermore, due to the lack of a blood–brain barrier, both AVPs might access CVOs after systemic administration [9,50]. Limited evidence indicates that local administrations of AVP into the AP produce both excitatory and inhibitory responses mediated by V1Rs [50]. Furthermore, AVP administered directly into the AP [12] or into the cerebral ventricles [94] may decrease respiratory rate in rats. In contrast, intracerebroventricular administration of AVP had no effect on respiratory rate in a macaque monkey [95]. Nonetheless, in all those studies, pulmonary ventilation was not evaluated.

There are several limitations in our study. Firstly, all measurements were carried out under urethane anesthesia, which may diminish some regulatory effects of vasopressin. Hemodynamic and ventilatory parameters, as well as reflexes, evaluated under urethane anesthesia, deviate from those observed in awake rats. Nonetheless, available evidence suggests less pronounced effects of urethane anesthesia on cardiorespiratory regulation than other anesthetic agents used in experimental studies [42,44,96]. Further evaluation of cardiorespiratory responses to AVP in conscious animals should be more relevant physiologically. Secondly, in our experimental design, we could not fully dissect the contribution of vasopressin to respiratory effects from the impact of arterial baroreflex activation on pulmonary ventilation. An increase in arterial blood pressure is known to inhibit respiratory activity [86,87,88,89]. Comparison to equipressor doses of pure vasoconstrictor drugs (e.g., phenylephrine) could provide additional information on the extent of baroreflex-mediated inhibition of pulmonary ventilation. Alternatively, local administrations of low doses of AVP into the carotid body would eliminate its systemic effects. Thirdly, successful recordings of femoral artery blood flow were obtained in a limited number of animals; thus, estimation of changes in vascular resistance of the hindlimb during the arterial chemoreflex and in response to exogenous vasopressin was not possible. Finally, further studies are needed to determine the role carotid bodies and CVOs play in the cardiorespiratory effects of vasopressin.

## 5. Conclusions

Our study shows that pressor and respiratory response to vasopressin is augmented in hypertensive SHR rats. The cardio-respiratory effects of vasopressin appear to be mediated by V1aRs, as blockade of the receptors prevents changes in hemodynamic and respiratory parameters. Denervation of carotid bodies diminishes pressor response to AVP in normotensive rats and prevents AVP-induced decrease in pulmonary ventilation in hypertensive rats. These findings reveal the role of carotid chemoreceptors in arterial blood pressure and respiratory effects of intravenously administered vasopressin in hypertensive SHR and normotensive WKY rats. By revealing the role of the carotid body and V1aRs in the pressor and respiratory effects of vasopressin, our study provides a new framework for developing targeted therapies aimed specifically at the carotid bodies and vasopressin V1a receptors for managing hypertension and related respiratory issues.

## Figures and Tables

**Table 1:** Baseline hemodynamic and respiratory parameters in spontaneously hypertensive (SHR) rats and normotensive Wistar Kyoto (WKY) control rats.

Parameter	SHR	WKY	*p*-Value
MABP (mm Hg)	90.3 ± 14.2	63.6 ± 5	<0.001
HR (beats/min)	297.4 ± 30.8	235.8 ± 44	<0.001
FABF (mL/min)	1.2 ± 0.7	1.6 (1, 2.2)	0.180
MV (mL/min)	289 ± 45	203.2 ± 38.5	0.007
RR (breaths/min)	59.2 ± 13.9	89.8 (66.7, 112.9)	0.002
ETCO2 (%)	4.4 ± 0.3	4.5 ± 0.5	0.373

## Data Availability

Research data is available upon reasonable request.

## Abbreviations

The following abbreviations are used in this manuscript:

AP	area postrema
AVP	arginine vasopressin
BP	blood pressure
CB/CBs	carotid body/carotid bodies
CBX	carotid body denervation
CVOs	circumventricular organs
ECG	Electrocardiogram
ET-1	endothelin 1
ETCO_2_	end-tidal carbon dioxide
FABF	femoral artery blood flow
GLP-1	glucagon-like peptide 1
HR	heart rate
KCN	potassium cyanide
MABP	mean arterial blood pressure
MV	minute ventilation
OVLT	organum vasculosum laminae terminalis
RR	respiratory rate
SFO	subfornical organ
SHR	spontaneously hypertensive rat(s)
V1aR	vasopressin type 1a receptor
V1aR ANT	antagonist of V1aR
WKY	Wistar Kyoto rat(s)
